# Performance Analysis of a Head and Eye Motion-Based Control Interface for Assistive Robots

**DOI:** 10.3390/s20247162

**Published:** 2020-12-14

**Authors:** Sarah Stalljann, Lukas Wöhle, Jeroen Schäfer, Marion Gebhard

**Affiliations:** Group of Sensors and Actuators, Department of Electrical Engineering and Applied Physics, Westphalian University of Applied Sciences, 45877 Gelsenkirchen, Germany; lukas.woehle@w-hs.de (L.W.); jeroen.schaefer@uni-bremen.de (J.S.); marion.gebhard@w-hs.de (M.G.)

**Keywords:** assistive technology, motion sensors, eye tracker, MARG, tetraplegia, Fitts’ Law, robot control, cursor control

## Abstract

Assistive robots support people with limited mobility in their everyday life activities and work. However, most of the assistive systems and technologies for supporting eating and drinking require a residual mobility in arms or hands. For people without residual mobility, different hands-free controls have been developed. For hands-free control, the combination of different modalities can lead to great advantages and improved control. The novelty of this work is a new concept to control a robot using a combination of head and eye motions. The control unit is a mobile, compact and low-cost multimodal sensor system. A Magnetic Angular Rate Gravity (MARG)-sensor is used to detect head motion and an eye tracker enables the system to capture the user’s gaze. To analyze the performance of the two modalities, an experimental evaluation with ten able-bodied subjects and one subject with tetraplegia was performed. To assess discrete control (event-based control), a button activation task was performed. To assess two-dimensional continuous cursor control, a Fitts’s Law task was performed. The usability study was related to a use-case scenario with a collaborative robot assisting a drinking action. The results of the able-bodied subjects show no significant difference between eye motions and head motions for the activation time of the buttons and the throughput, while, using the eye tracker in the Fitts’s Law task, the error rate was significantly higher. The subject with tetraplegia showed slightly better performance for button activation when using the eye tracker. In the use-case, all subjects were able to use the control unit successfully to support the drinking action. Due to the limited head motion of the subject with tetraplegia, button activation with the eye tracker was slightly faster than with the MARG-sensor. A further study with more subjects with tetraplegia is planned, in order to verify these results.

## 1. Introduction

According to international incidence data, 250,000–500,000 people per year suffer a spinal cord injury (SCI) worldwide. SCIs can caused by accidents or by diseases such as multiple sclerosis, muscular dystrophy, or tumors. The loss of motor function of the arms and legs due to SCI is described as tetraplegia [1]. People with tetraplegia are dependent on support in their everyday life and work and require comprehensive home care. This support is provided by assistants. With appropriate assistance robots, people with tetraplegia would be able return to work, as has been shown with the FRIEND system at Bremen University Library. This system supports a subject with tetraplegia in the task of retrospectively cataloging books. Gräser et al. showed that interaction and collaboration between the user and system result in a higher success rate than a completely autonomous system [2]. The inclusion of the superior visual senses of humans might be an advantage when controlling a robot or technology. Therefore, it is useful to combine multiple sensor modalities, in order to control an assistive technology in the best possible way. The research and development of assistive technologies and robots in the field of activities of daily living (ADL) are also important for people with tetraplegia, in order to improve their quality of life, as assistance robots with interactive basic skills provide more independence for people with tetraplegia and relief for carers. These ADLs include self-determined eating and drinking. Different assistive technologies are available to support people with limited mobility in eating and drinking. However, residual mobility in the arms, hands, and upper body is necessary to control these products, and thus people without such residual mobility are not able to use them [3]. Hands-free control is a requirement for assistive systems for these people. Several different hands-free Human–Machine Interface (HMI) concepts and assistive devices for people with tetraplegia are reviewed in [4]. In the following section, examples of controlling assistive technologies and robotics with different sensor modalities are presented.

### 1.1. State-of-the-Art on Sensor Modalities for Control Interfaces

Assistive technologies are controlled through different input modalities, e.g., speech, EEG (Electroencephalography), EMG (Electromyography), or movements of the head, eyes, or tongue. Hochberg et al. presented an example of controlling a robot using neural activity [5]. In their work, the signals of motor cortex neurons of subjects with tetraplegia were used to control a robot arm and perform grasp tasks. The neural signals were recorded using a microelectrode array implanted in an area of the motor cortex of the subject. Therefore, this method is an invasive method to control robots. The implanted electrode array is a limitation and thus the method is only usable for a small group of people. Another sensor modality is EMG, which involves the measurement of muscle activity and can be used to control an assistive technology. In [6], subjects with tetraplegia controlled a wheelchair by only activating their posterior auricular muscles and were able to complete an obstacle course. This provides subjects with tetraplegia more independence in the field of mobility. To activate the posterior auricular muscles, the subjects had to complete a preliminary training using software-based training and visual feedback. This method is therefore only usable with prior several days of training. Furthermore, the system was tested on a wheelchair in two-dimensional space and not on a robotic arm in three-dimensional space.

To control a robot in three-dimensional space, Alsharif [7] used the gaze direction, gaze gestures, and states of the eyes. For measuring the gaze direction and states of the eyes, the SensoMotoric Instruments (SMI) Eye Tracking Glasses (ETG) was used. Since the SMI ETG is worn as normal glasses, spectacle wearers were unable to use it. This reduced user acceptance. One method for gaze control is eye tracking, especially for people with limited mobility, e.g., to control a cursor on a computer screen/touchpad and trigger actions. In this case, the eye offers an independent channel of input. The user can produce a lot of eye movements without obvious symptoms of fatigue in this modality [8]. A fundamental obstacle while using eye tracking is the so-called “Midas Touch” problem. The saga about King Midas is used to describe this problem. Initially, it appears to be an advantage that the user controls things and interacts with objects simply by looking at them (i.e., without the help of hands). However, they then become no longer able to use their eyes for “normal vision”, as everything the user looks at is clicked on or interacted with. The distinction of whether the eyes of the user are used for sensory perception of the environment or for object interaction is not possible [9]. An option to minimize the “Midas Touch” problem is the so-called “Dwell Time”. In this case, the object/button has to be fixated for a certain time, in order to trigger an action. For this method, it is important to select an appropriate Dwell Time: If the Dwell Time is too short, unintended actions are triggered quickly, while, if the Dwell Time is too long, the user becomes impatient and fixation of the button might get lost. Thus, triggering actions may take too long [8].

Besides eye movements, head movements can also be used as a control modality. An example for an assistive robot system which uses head motion control is the Adaptive Head Motion Control for User-friendly Support (AMiCUS) system [10]. Using this system, subjects with tetraplegia could successfully perform different pick and place tasks [11]. The head motions are recorded using Micro-Electro-Mechanical System (MEMS) Magnetic Angular Rate and Gravity (MARG)-sensors. MARG-sensors consist of a three-axis accelerometer, a three-axis gyroscope, and a three-axis magnetometer. Through the combination of these sensors, the absolute orientation can be calculated. MEMS sensors are very small micromechanical structures and associated control elements on one chip (i.e., a System on a Chip, SOC) [12]. The advantage of these sensors is their very compact design and size. They may be placed almost anywhere and may also be attached, for example, to a headset without disturbing the user.

### 1.2. State-of-the-Art on Evaluation of Sensor Modalities

For the evaluation of sensor modalities, the analysis of the time required for given tasks and the error rate is a proven method. To investigate the performance and usability of sensor modalities, the so-called “Fitts’s tapping tasks” are typically performed. The Fitts’s Law paradigm is a standard test to evaluate the effectiveness and efficiency of input devices [13]. Zhang and MacKenzie used the Fitts’s Law paradigm to examine different eye tracking techniques [14]. Motion sensors were also evaluated using the Fitts’s Law test [15]. To evaluate the subjective workload when using control interfaces, the NASA Task Load Index (NASA-TLX) questionnaire is used worldwide [16]. This questionnaire measures the subjective workload in six different subscales. For the assessment of the usability, individually created questionnaires are used. The Likert scale is often used in these questionnaires as it is one of the most reliable instruments for measuring opinions and behavior [17]. Different statements could be evaluated and the participant should indicate whether he or she “fully agrees” to “fully disagrees” with these statements.

### 1.3. Previous Work

Some elements of the robot control concept related to our previous work on the AMiCUS system [10] have been adapted and used in this work. Therefore, the AMiCUS system is presented in more detail in the following. The AMiCUS system uses head motion and head gestures to continuously control a robot arm with a gripper. The three Degrees of Freedom (DOFs) of the head are mapped to the seven DOFs of the robot arm and the robot movements are divided into different robot groups. The head movements are used to continuously control the robot motion. Switching between robot control groups is enabled by head gestures. While using the AMiCUS system, the user chooses between two control modes, the so-called *robot mode* and the *cursor control mode*. During the *robot mode*, direct three-dimensional robot motions are possible. During the *cursor control mode*, the user moves a cursor on a screen and performs actions such as starting a calibration routine or selecting a robot movement group. However, the evaluation of head gestures with able-bodied subjects and subjects with tetraplegia indicated problems, in terms of fatigue [11]. To overcome these problems, a multimodal unit which combines head and eye motions for controlling a robot and a cursor is presented here. The monomodal concept of control is extended, by the human visual system, to a multimodal system. It was decided to extend the head motion interface by eye motions, because this sensor modality is generally suitable for controlling a robot, as described in Section 1.1. However, an eye tracker should be used, which may also be worn by people who wear glasses. Furthermore, the eye tracking is a non-invasive method and can be used without prior sensor implantation. Both MARG-sensors and some eye trackers are compact in their design and size. This allows the development of a mobile, compact, and low-cost multimodal sensor control unit. To minimize the Midas Touch problem, which occurs during eye tracking, the option Dwell Time was implemented (see Section 2.3). The purpose of this work is a performance analysis of the control unit. The two modalities are examined in the context of discrete and continuous control. Therefore, two different tests were performed using the modalities alternately. The multimodal control unit was tested in a scenario relevant to supporting subjects with tetraplegia in their everyday lives. In the use-case scenario, both modalities were used simultaneously and we examined how comfortably and intuitively a subject was able to grasp a cup with the control unit and bring the cup to their mouth. The following section describes the multimodal control unit. The experimental design, setup, and procedures of different tests to evaluate the control unit are presented. Then, the results of the different tests are shown and discussed. In the final section, our conclusions are given and the future work is explained.

## 2. Materials and Methods

In this section, the multimodal control unit is introduced and the performed tests and recorded parameters for analyzing the performance of the control unit are described. In this work, different control modes were used: *discrete control, continuous cursor control, and continuous robot control*. The discrete control mode describes the event-based control. In this mode, interaction elements were activated in the form of buttons on a Graphical User Interface (GUI). In the continuous cursor control mode, a cursor was moved on a screen (i.e., in two dimensions). In discrete control mode as well as in continuous cursor control mode, the control was performed either with the MARG-sensor or with the eye tracker. In continuous robot control mode, the robot arm was continuously moved in three-dimensional space. For this control mode, only head motions were used. The control with head motions has proven to be robust during previous work [10]. In *Activation of Buttons Test* (see Section 2.3.1), we examined which modality was better suited to activate different buttons. In *Continuous Cursor Control Test* (see Section 2.3.2), we tested which modality was better suited to moving the cursor on the screen. The *use-case scenario* (see Section 2.3.3) assessed how comfortably and intuitively a subject was able to grasp a cup and lead it to their mouth when the subject used the proposed control unit to control the robot arm. In the use-case, all three control modes were used to perform the task.

### 2.1. Subjects

In the present study, three tests were implemented to analyze the performance of the control unit. Twelve able-bodied persons and one subject with tetraplegia performed the three tests as a proof of concept for the proposed control interface. The data of two able-bodied persons were not included in the analysis, as they were not able to finish all three tests due to technical problems. The able-bodied subjects had no known limitations, in terms of head movement. Five of them were male and five female. They were recruited through flyers and announcements listed on the university website. Their age ranged from 20 to 56 years (mean ± standard deviation (SD): 28.20 ± 10.70 years). Three of the subjects wore glasses and one subject wore contact lenses. All subjects gave their written consent to the experiment and were instructed in detail about the procedure. This study was approved by the ethics committee of the German Association of Social Work (DGSA).

### 2.2. Experimental Setup

**Figure 1:**
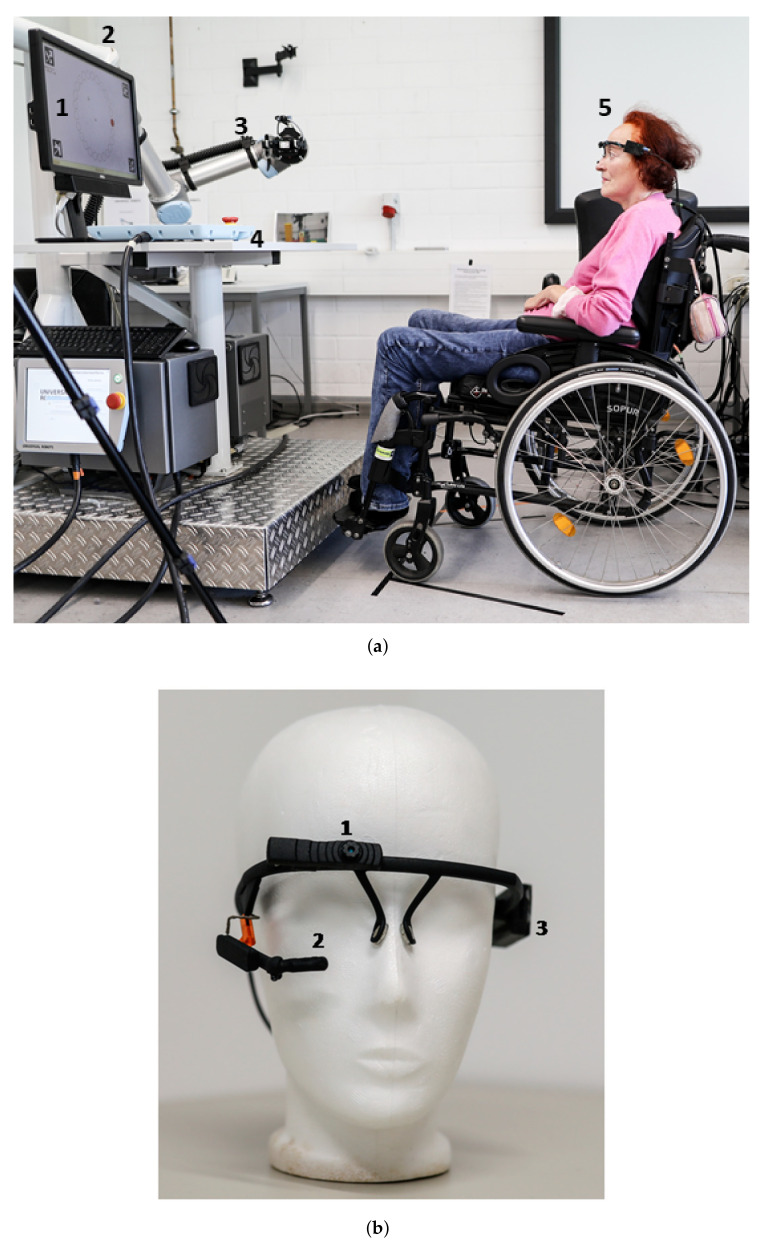
(**a**) Experimental setup with screen to display the GUIs for the tests (1), metal torso (2) on which the robot arm with gripper (3) is mounted, table (4), and subject with the multimodal control unit (5); and (**b**) the multimodal control unit consists of an eye tracker Pupil Core (worldcamera (1) and eye camera (2)) and a MARG-sensor (3).

Figure 1a shows the experimental setup. The used robot arm was a UR5 robot by Univeral Robotos [18] with an adaptive 2-Finger gripper Robotiq 85 [19]. A 23 inch screen was used to display the GUIs for the different tests. The subjects sat in front of the metal platform and the distance between subjects and the screen was 90 cm. The working area of the robot was set between its home position and the table area. During the tests, the subjects wore the multimodal control unit presented in Figure 1b. A MARG-sensor [20,21] was mounted in a printed case on the frame of an eye tracker. The sample rate of the MARG-sensor was 100 Hz. A monocular eye tracker headset (Pupil Core from Pupil Lab) was used [22]. The eye tracker (ET) tracked the pupil of the eye to display the person’s gaze point on a screen, in order to follow eye movements. The frame rate of the world camera was 30 Hz at a resolution of 1920 × 1080 pixels, and that of the eye camera was 90 Hz at a resolution of 400 × 400 pixels.

### 2.3. Procedure

The experiment consisted of a trial phase and three tests. The trial phase was the first part of the experiment and gave the subjects the possibility to try out the system. The next part was a test examining discrete control using the multimodal control unit. During this test, the subjects had to activate different buttons. To evaluate the continuous cursor control, the subjects performed a two-dimensional Fitts’s Law paradigm in the third part of the experiment. In the last part, the multimodal control unit was used to perform a drinking task. In this use-case scenario, all three control modes were used: discrete control (activating buttons), continuous cursor control (continuous two-dimensional moving of a cursor on the screen), and continuous robot control (continuous three-dimensional moving of the robot arm).

Before starting with the trial phase, the subjects read the participant information and signed the declaration of content. Then, the multimodal control unit was placed on and fixed with a eyewear strap on the users head, in order to prevent movements of the eye tracker. The eye camera was adjusted to track the pupil of the subject’s eye and the world camera captured the monitor screen on which the GUIs for the tests were represented. The next step was the calibration of the eye tracker, as described in [22], to establish a mapping from pupil to gaze coordinates. In the trial phase, the subjects tested the system with the multimodal MARG-sensor and eye tracker control unit. In the continuous robot control mode, head motions were used. The three DOFs of the head were mapped to seven DOFs of the robot arm with gripper, as described in [23]. Calibration of the MARG-sensor for the robot control and cursor control was performed using the calibration routines described in [10]. Before starting each test, the subjects read a detailed set of written instructions about the task. Between the different tasks, the subjects had the possibility to take a break. The eye tracker calibration was repeated for every test.

**Figure 2:**
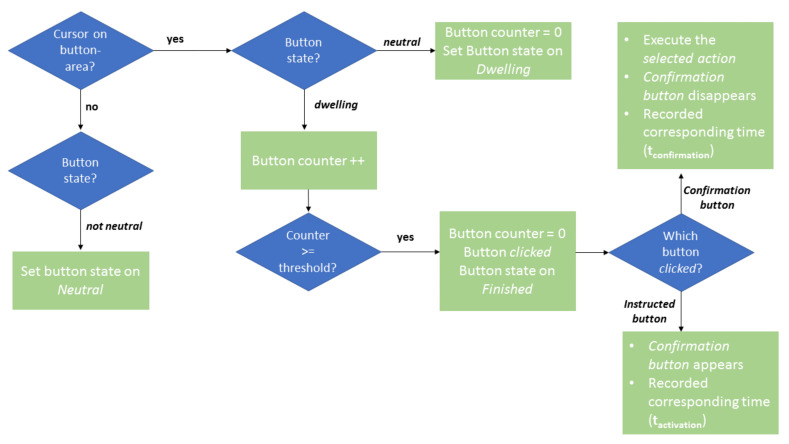
Flowchart of the Dwell button implementation in the GUI used for the *Activation of Buttons Test* and the *Use-Case Test*.

For the *Button Activation Test* (Section 2.3.1) and in the *use-case scenario* (Section 2.3.3), a GUI with Dwell buttons was used. Figure 2 shows the activation mechanism of these buttons. The Dwell button type was chosen to reduce the Midas Touch problem. A certain Dwell Time was used, specifying that the subject had to fixate on an object or button for a certain duration before an action was triggered [8]. For this study, a Dwell button with a corresponding confirmation button was used, which was also implemented as a Dwell button. This allowed for double checking before a button was activated to trigger an action. While the user moved the cursor on the screen, it was checked whether the cursor was on a button area. If not, the status of the button was set to neutral. If yes, it was checked if the status was neutral or dwelling. If the status was neutral, it was then set to dwelling. If status was dwelling, the counter was increased. If a threshold was exceeded, the counter was reset to zero, the button was activated, and the button status was set to finished. Then, it was checked which button was activated (i.e., the instructed button or the corresponding confirmation button). If the instructed button was clicked, the corresponding confirmation button was displayed on the GUI. The user then had to activate the confirmation button. If the confirmation button was clicked, the selected action was performed and the confirmation button disappeared again. The threshold for both modalities was 50 samples.

#### 2.3.1. Activation of Buttons Test

**Figure 3:**
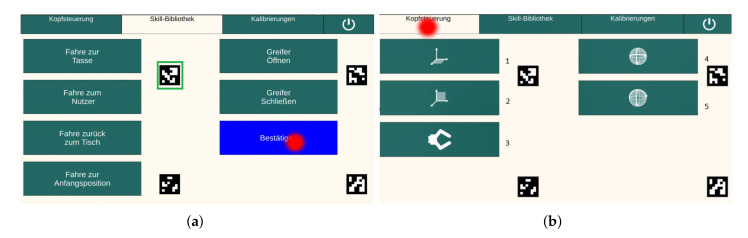
Test 1: (**a**) GUI with the six buttons of the skill library and confirmation button (blue rectangle), green rectangle = aruco marker, red point = cursor controlled by either eye or head movements on the screen; and (**b**) GUI with the five buttons of the head robot control, red point = cursor controlled by either eye or head movements on the screen, 1 = hplane, 2 = vplane, 3 = gripper, 4 = orient1, and 5 = orient2.

This test examined the discrete control through the activation of buttons on a GUI. Eleven different buttons were available for activation: six buttons in the skill library group (see Figure 3a) and five buttons in the head control group (see Figure 3b). A skill library was created that allowed the user to move the robot to fixed positions without having to switch to the head control. The buttons in the skill library were based on the use-case scenario. The drinking process was divided into different sections: picking up the cup, moving the cup towards the user, drinking, putting the cup down, and moving the robot in its home position. Therefore, four buttons for fixed robot positions (*cup*, *user*, *back to the table*, and *starting position*) and two buttons to open and close the gripper (*open* and *close*) were implemented. By activating the buttons in head control group, the user continuously controlled the robot arm. For controlling the robot, the three DOFs of the head were mapped to seven DOFs of the robot arm. Due to the different number of DOFs, the movements of the robot were divided into different groups. These different groups were implemented as the five buttons in the head control group (*hplane*, *vplane*, *gripper*, and *orient1 and orient2*). This implementation was proven to be most useful in the work of Rudigkeit et al. [10], and it was thus adopted for this work. The task of the subjects was to activate different buttons, according to the instructions of the experimenter. The instruction sequence was structured in such a way that each of the eleven buttons had to be activated three times and no button was activated directly twice in a row. A button was activated by moving the cursor on the button area and holding it there for some time (see Figure 2). After the button click, a blue confirmation button then appeared (see Figure 3a). The cursor also had to be moved to this button and held there for some time (see Figure 2). It was only after the confirmation button had been clicked that the activation of the button was successful and the selected action was executed. The corresponding times of the instructed button click and the confirmation button click were recorded as $t_{activation}$ and $t_{confirmation}$, respectively. The test was performed twice, once with the eye tracker and the other with the MARG-sensor. When using the eye tracker, the cursor was controlled with eye motions, while, when using the MARG-sensor, the cursor was controlled with head motions. To prevent learning effects, the starting order of the modalities was randomized.

#### 2.3.2. Continuous Cursor Control Test

**Figure 4:**
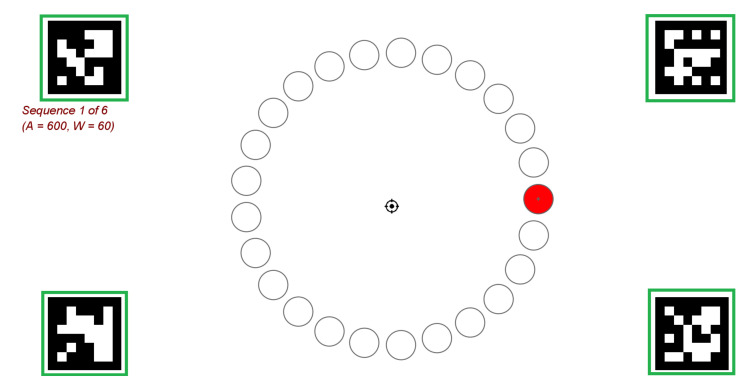
Fitts’s Law Test: Example of a screen output Condition No. 1, red marked circle = starting position, green rectangles = aruco marker.

During the continuous cursor control test, the subjects performed the two-dimensional Fitts’s Law paradigm, according to ISO 9241-9, as described in [13,14]. The Fitts’s Law test is a standard test to evaluate the performance of input devices (except keyboard devices). The software used in this experiment was based on the software developed by MacKenzie [24]. An example of a screen output is shown in Figure 4. On the screen, 25 circles (targets) are displayed, arranged in a circle. One circle is alternately marked in red. The starting position is the red marked circle in Figure 4 and, to perform the task with the eye tracker, four aruco markers are added. The task was to move the mouse cursor to the red marked circle and hold the mouse cursor there until the next red marked circle at the opposite site appeared. A Dwell Time of 200 ms was set for target activation. The entire test consisted of several sequences, in which both the diameter of the targets (width W) and the distance between the centers of two opposite targets (amplitude A) varied. A sequence contained 25 trails (25 targets/circles where the mouse cursor had to move to). For the distance, 600 and 800 pixels were chosen. The targets had diameters of 60, 80, and 100 pixels. In total, there were six different conditions. Between the procedures, the subjects were offered the possibility to take a short break. Each condition was performed with the eye tracker (mouse cursor is moved by eye motions) and the MARG-sensor (mouse cursor is moved by head motions). To prevent learning effects, the starting order of the modalities was randomized. Furthermore, the option *“Randomize Target Conditions”* was selected in the software, such that the sequence of the six conditions was randomized for each subject. Table 1 shows the chosen distances and diameters of the targets for the six conditions.

#### 2.3.3. Use-Case Test

**Figure 5:**
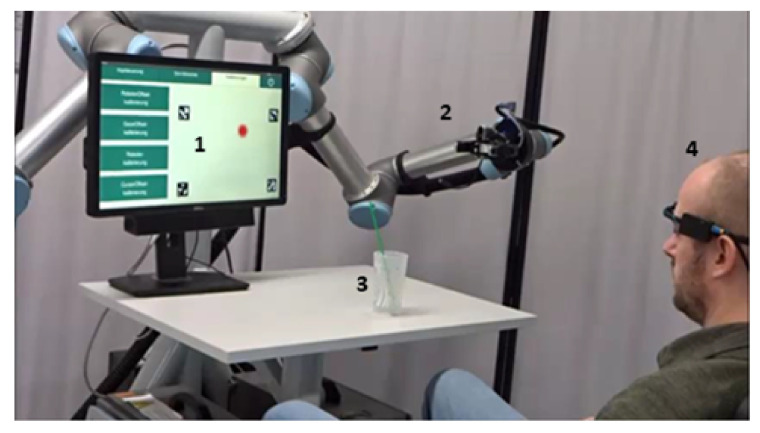
Experimental setup of use-case scenario with GUI to select the buttons (1), robot arm with gripper (2), cup with straw (3), and subject with the multimodal control unit (4).

The experimental setup of the use-case is presented in Figure 5. In this test, the eye tracker was used to continuously control the cursor on the screen and for discrete control; that is, for button activation. The robot arm was moved to fixed positions by activating the buttons in the skill library (see Figure 3a). The robot arm was able to be moved to the cup, to the user, back to the table, or to the home position. It was also possible to open and close the gripper. Head motions were used to continuously control the robot arm. Using the buttons of the head robot control (see Figure 3b), the robot could be moved in the horizontal plane (back and forth, left and right) and in the vertical plane (up and down, left and right). Furthermore, it was possible to change the orientation of the gripper and to open and close it. The task in the use-case was to grasp the cup using of the robot arm’s gripper and to move it close to the user, such that drinking through a straw was possible. Then, the cup had to be placed back on the table and the robot arm had to be moved to its home position. The experimenter told the subject when a button of the skill library should be activated to move the robot arm to a fixed position, or when the buttons in the head control system should be used to move the robot independently. The following sequence was used:

1. Gripper is moved close to the cup position using the button *Tasse* (skill library)
2. Gripper is moved to the cup using the buttons of the *Kopfsteuerung* (robot control)
3. Gripper is closed using the button *Greifer schließen* (skill library)
4. Gripper is moved close to the user using the button *Nutzer* (skill library)
5. Gripper is moved to the final drinking position using the buttons of the *Kopfsteuerung* (robot control)
6. User drinks
7. Gripper is moved back to the table using the button *zurück zum Tisch* (skill library)
8. Gripper is moved using buttons of the *Kopfsteuerung* (robot control), such that the cup is placed on the table
9. Gripper is opened using the button *Greifer öffnen* (skill library)
10. Gripper is moved back to the home position using the button *Anfangsposition* (skill library)
11. Program is closed using the *shut-down button*

### 2.4. Evaluation Criteria

#### 2.4.1. Activation of Buttons Test

Both objective and subjective parameters were measured to evaluate the multimodal control unit in the context of discrete control. Objective parameters were the time needed to activate a button and the error rate. The activation time was defined as the time from the click of the given button to the click of the confirmation button (*t* [s] = $t_{confirmation}$ − $t_{activation}$). An activation was rated as false if a wrong button was activated (type 1 error) or if the subject was too slow in activating the button (type 2 error; blue confirmation button disappears automatically after 5 s). If the activation is rated as type 2 error, the button was not activated at all and no action was triggered. The rating was done by the experimenter while the subjects performed the task. Incorrect activations were repeated by the experimenter, such that 33 correct activations were recorded per subject (three per button). The mean value per button was calculated from the three activation times for each button. In the group of the able-bodied subjects, 355 trials were performed (330 correct trials and 25 incorrect trials). The subject with tetraplegia performed 38 trials (33 correct trials and 5 incorrect trials). Due to the different sample rates of the MARG-sensor and eye tracker, it took different amounts of time until the threshold (which was defined in terms of samples) was exceeded. Due to COVID-19, it was not possible for the subjects to repeat the button activation test in which the activation mechanism of the Dwell buttons was implemented with a threshold in seconds. To compare the two modalities, the acquired time values had to be corrected. For this purpose, the times needed for the MARG-sensor and the eye tracker to exceed the threshold were determined. As explained in Figure 2, a counter was increased, if the status of a button was set to *dwelling*. This time point $t_{dwelling}$ was recorded. If the threshold was exceeded, the button status was set to *finished*. This time point $t_{finished}$ was also recorded. The difference between $t_{finished}$ and $t_{dwelling}$ indicated how many seconds it took until the threshold value was exceeded ($t_{thresholdexceeded}$). Members of our research group activated the confirmation button hundred times with both modalities and the average value of $t_{thresholdexceeded}$ was calculated per modality. The difference between the average values of $t_{thresholdexceeded}$ of the MARG-sensor and $t_{thresholdexceeded}$ of the eye tracker was calculated and was 1.02 s. This value was used to correct the activation times. Immediately after performing the task, the subjects had to fill out the NASA-TLX questionnaire, which measures the subjective perceived workload. This questionnaire was used as a subjective parameter. Mental, physical, and temporal demands, as well as performance, effort, and frustration were recorded using six sub-scales. The original version of the questionnaire [16] consists of two parts: in the first part, the six sub-scales are rated independently, while, in the second part, the scales are compared and weighted in pairs, based on their contribution to the perceived workload. Then, the total workload is calculated using the weighting factors. In this study, the most commonly modified version of the NASA-TLX, the Raw Task Load Index (RTLX), was used [25]. In the case of the RTLX, the weighting is eliminated, resulting in one rating score per scale.

#### 2.4.2. Continuous Cursor Control Test

To evaluate the continuous control, two different objective parameters were calculated using the Fitts’s Law software [24], according to the standard procedures of ISO 9241-9. The parameters were the throughput and error rate, which were calculated for each sequence of the six conditions. The movement time (MT, measure of speed [s]) and the effective index of difficulty (${\mathrm{ID}}_{e}$, measure of accuracy [bits]) were used to determine the throughput (TP, measure of the performance [bps]), as shown in Equation (1).

(1) $$TP=ID_{e}/MT.$$

**Figure 6:**
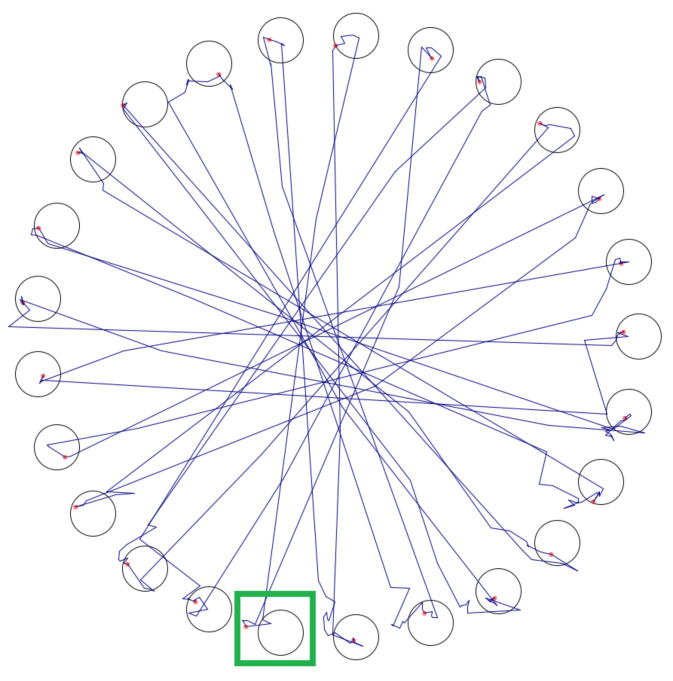
Example of the path of the mouse cursor taken by the subject during the task, green rectangle = target which is rated as error.

The error rate was calculated using the focus of the detected points, which were approached until a target was actually activated. Figure 6 shows an example of the path of the mouse cursor taken by a subject during the task. One target (green rectangle) was rated as an error because the focus of the detected points was not within the target (small red dot). In this example, the error rate was 4.00% (1 of 25 targets).

#### 2.4.3. Use-Case Test

In the use-case scenario the subjects had to perform a sequence with eleven steps to solve the task (see Section 2.3.3). During the instructions of the experimenter, it was checked whether the correct buttons were activated and thus the correct steps and actions were performed. These observations were used to determine the completion rate of the task as an objective parameter to evaluate the control unit in the use-case. As subjective parameter for evaluating the multimodal control unit in the use-case, the subjective perceived workload was measured using the NASA-TLX [16] after completing the task. Mental, physical, and temporal demands, as well as performance, effort, and frustration, were recorded using six sub-scales. In this study, the most commonly modified version of the NASA-TLX, the Raw Task Load Index (RTLX), was used [25]. In addition, the subjects filled out a questionnaire in which the control unit was evaluated. The statements of this questionnaire are presented in Table 2, which were divided into three parts relating to cursor control, robot control, and general control (see Section 3.3). For the rating, a Likert scale [17] with values ranging from *1 “I do not agree at all”* to *5 “I totally agree”* was used.

## 3. Results

### 3.1. Activation of Buttons Test

In the following section, the results of the button activation test are presented. For the able-bodied subjects, the activation time averaged over all eleven buttons was 1.67 ± 0.06 s (SD) for the eye tracker and 1.53 ± 0.11 s (SD) for the MARG-sensor. The difference of the activation time between modalities was not significant (*p* > 0.05). The able-bodied subjects produced significantly more (*p* = 0.002) type 2 errors (too slow in activating the button) with the eye tracker (error rate: 5.76 ± 3.90% SD) than with the MARG-sensor (error rate: 0.30 ± 0.30% SD). For the type 1 errors (wrong button was clicked) there was no significant difference (*p* > 0.05) between the two modalities (ET error rate: 0.61 ± 0.40% SD, MARG-sensor error rate: 0.91 ± 0.46% SD). For the subject with tetraplegia, the activation time averaged over all eleven buttons was 1.90 s for the eye tracker and 2.58 s for the MARG-sensor.

**Figure 7:**
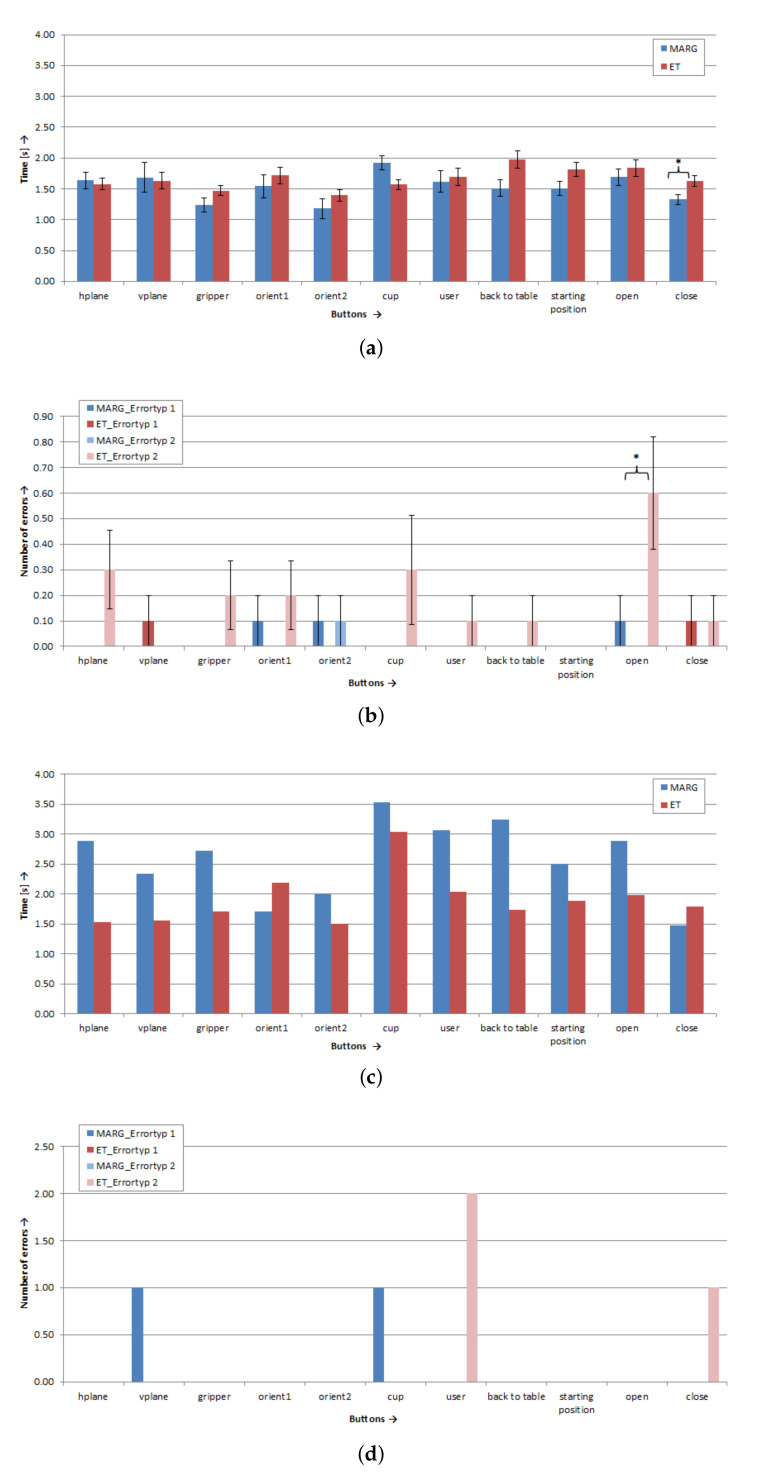
Results of test button activation for the eleven buttons: (**a**) Able-bodied subjects (*n* = 10), mean value of activation time ± standard error (SE); (**b**) able-bodied subjects (*n* = 10), mean value of number of errors ± SE; (**c**) subject with tetraplegia (*n* = 1), activation time values; and (**d**) subject with tetraplegia (*n* = 1), number of errors. * Marks sig. differences (*p* < 0.05).

Figure 7 shows an overview of the results of the button activations. To compare both modalities (eye tracker and MARG-sensor) in the able-bodied subjects, paired sample t-tests were used for each button. The mean activation times for the able-bodied subjects are presented in Figure 7a. A significant difference between the activation times of both modalities was found for button *close* (*p* = 0.022). The subjects activated this button with the MARG-sensor significantly faster (1.33 ± 0.26 s SD) than with the eye tracker (1.63 ± 0.26 s SD). For the other ten buttons, there were no significant differences between the activation times of the eye tracker and MARG-sensor (*p* > 0.05). For the number of errors, the difference between the modalities for button *open* for type 2 errors was significant (see Figure 7b). For this button, the subjects made significantly more type 2 errors with the eye tracker than with the MARG-sensor (*p* = 0.024). However, no significant difference was found between the number of errors (of type 1 and type 2) for the two modalities in the other ten buttons (*p* > 0.05).

In Figure 7c, the activation times for the subject with tetraplegia for the eleven different buttons are presented. For nine of the eleven buttons, the subject with tetraplegia needed more time to activate these buttons with the MARG-sensor than with the eye tracker. In general, the activation times of the subject with tetraplegia for the MARG-sensor were longer than the activation times of able-bodied subjects with the MARG-sensor. The subject with tetraplegia produced a similar number of errors with both modalities. The subject made three errors with the eye tracker and two errors with the MARG-sensor (see Figure 7d). However, the results show that the subject with tetraplegia only made type 2 errors with eye tracker. In these cases, the subject was too slow in activating the button. The subject produced only type 1 errors when using the MARG-Sensor. In these cases, the subject activated a wrong button.

**Figure 8:**
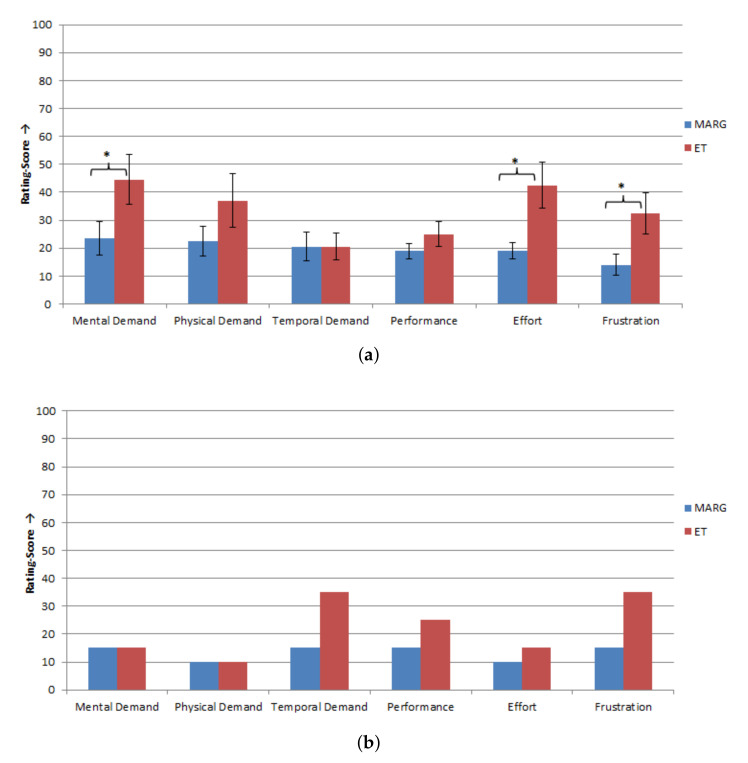
Results of the NASA-TLX questionnaire to measure the subjective perceived workload for Test 1, scale: 0 = low/good to 100 = high/poor: (**a**) able-bodied subjects (*n* = 10), mean values ± SE of the rating scores for the six sub-scales of the NASA-TLX; and (**b**) subject with tetraplegia (*n* = 1), values of the rating scores for the six sub-scales of the NASA-TLX. * Marks sig. differences (*p* < 0.05).

Figure 8 presents the results of the subjective perceived workload measured with the NASA-TLX questionnaire. For both modalities, the able-bodied subjects and the subject with tetraplegia rated the workload as low on all six sub-scales (all rating scores < 50, see Figure 8e,f). However, significant differences were found in the sub-scales *effort*, *frustration*, and *mental demand* between the modalities while activating the buttons for the able-bodied subjects (*p* < 0.05). They found the procedure with the eye tracker (effort: 42.50 ± 26.38 SD, frustration: 32.50 ± 23.24 SD) significantly more strenuous and more frustrating than with the MARG-sensor (effort: 19.00 ± 9.07 SD, frustration: 14.00 ± 11.98 SD). Activation of the buttons was significantly more mentally demanding while using the eye tracker (44.50 ± 28.33 SD) than using the MARG-sensor (23.50 ± 19.44 SD). For the subject with tetraplegia, performing with the eye tracker (35.00) was more frustrating than with the MARG-sensor (15.00). Furthermore, the temporal demand was higher while using the eye tracker (35.00) than using the MARG-sensor (15.00).

### 3.2. Continuous Cursor Control Test

In the following section, the results of the Fitts’s Law task are presented. For the able-bodied subjects, the throughput averaged over all six conditions was 2.01 ± 0.78 bps (SD) for the eye tracker and 2.24 ± 0.21 bps (SD) for the MARG-sensor. A paired sample t-test showed that the difference between the modalities was not significant (*p* > 0.05). The able-bodied subjects achieved the same performance with both modalities. In the complete Fitts’s Law task, the able-bodied subjects produced significantly more (*p* = 0.001) errors with the eye tracker (average error rate: 4.47 ± 2.29% SD) than with the MARG-sensor (average error rate: 0.73 ± 0.49% SD).

For the subject with tetraplegia, the throughput averaged over all six conditions was 0.85 ± 0.29 bps (SD) for the eye tracker and 1.19 ± 0.13 bps (SD) for the MARG-sensor. In the complete Fitts’s Law task, the error rate for the subject with tetraplegia was 8.00 ± 5.66% (SD) for the eye tracker and 1.33 ± 2.07% (SD) for the MARG-sensor.

**Figure 9:**
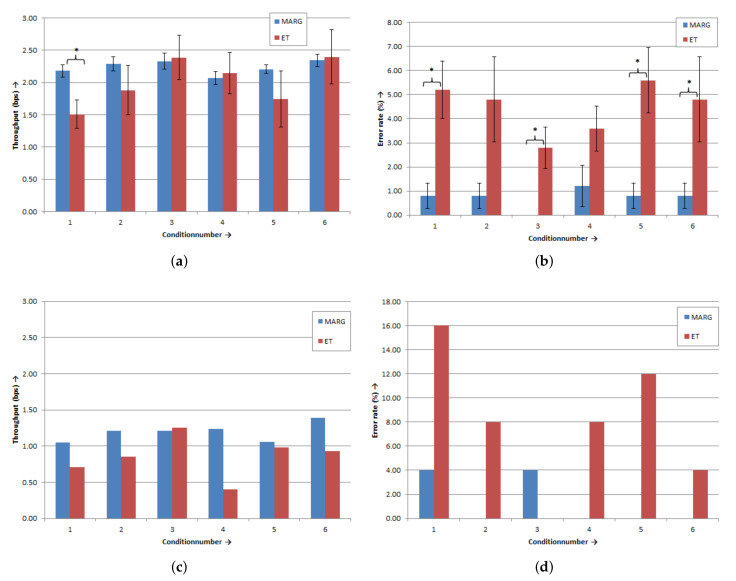
Results of the Fitts’s Law paradigm for the six conditions: (**a**) able-bodied subjects (*n* = 10), mean throughput values ± SE; (**b**) able-bodied subjects (*n* = 10), mean error rate values ± SE; (**c**) subject with tetraplegia (*n* = 1), throughput values; and (**d**) subject with tetraplegia (*n* = 1), error rate values. * Marks sig. differences (*p* < 0.05).

Figure 9 depicts an overview of the results of the Fitts’s Law task for the six different conditions. To compare both modalities for the group of the able-bodied subjects, we used paired sample t-tests for each condition. The determined mean throughputs for the able-bodied subjects are presented in Figure 9a. With the eye tracker and MARG-sensor, the subjects performed best in Conditions 3 and 6 (eye tracker: ${\mathrm{TP}}_{con3}$ = 2.39 ± 1.09 bps SD and ${\mathrm{TP}}_{con6}$ 2.40 ± 1.34 bps SD; MARG-sensor: ${\mathrm{TP}}_{con3}$ = 2.33 ± 0.39 bps SD and ${\mathrm{TP}}_{con6}$ = 2.34 ± 0.30 bps SD). For Condition 1 (A = 600 Pixel and W = 60 Pixel; see Table 1), a significant difference was found between the throughputs of the modalities (*p* = 0.033). The subjects showed, in this condition, a significantly higher throughput with the MARG-sensor (2.18 ± 0.31 bps SD) than with the eye tracker (1.51 ± 0.71 bps SD). However, there were no significant differences between the throughputs of the eye tracker and MARG-sensor for the other five conditions (*p* > 0.05). For the error rate, we found significant differences between the modalities in four of the six conditions (see Figure 9b). For Conditions 1, 3, 5, and 6, the subjects made significantly fewer errors with the MARG-sensor than with the eye tracker (*p* < 0.05).

Figure 9c shows the throughputs for the subject with tetraplegia for the six different conditions. The subject with tetraplegia demonstrated the highest performance with the MARG-sensor in Condition 6 (TP = 1.39 bps) and for the eye tracker in Condition 3 (TP = 1.25 bps). The subject with tetraplegia made more errors in each condition with the eye tracker than with the MARG-Sensor (see Figure 9d). With the eye tracker, the subject with tetraplegia made the most errors in Condition 1 (error rate 16.00%) and performed best in Conditions 3 and 6 (error rate 0.00% and 4.00%, respectively).

### 3.3. Use-Case Test

**Figure 10:**
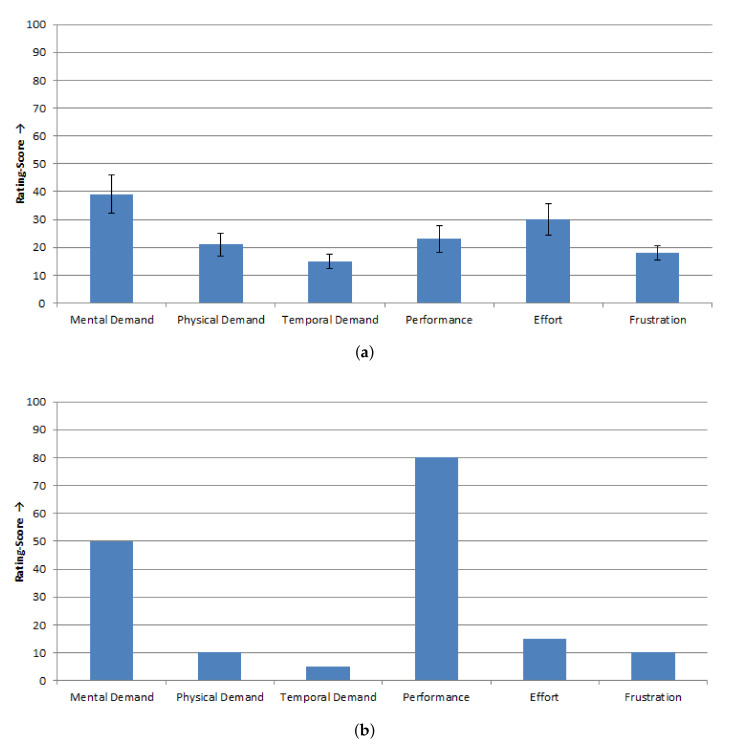
Results of the NASA-TLX questionnaire to measure the subjectively perceived workload for the use-case, scale: 0 = low/good to 100 = high/poor: (**a**) able-bodied subjects (*n* = 10), mean values ± SE of the rating scores for the six sub-scales of the NASA-TLX; and (**b**) subject with tetraplegia (*n* = 1), Values of the rating scores for the six sub-scales of the NASA-TLX.

In the use-case scenario, the subjects had to perform a sequence with eleven steps to solve the task. Nine of the ten able-bodied subjects and the subject with tetraplegia performed all eleven steps correctly. One subject performed ten of the eleven steps correctly and had to repeat one incorrect step. The completion rate of the use-case task for the able-bodied subjects was 90.00% and for the subject with tetraplegia 100.00%. For evaluation of the multimodal control unit in the use-case scenario, the NASA-TLX and a questionnaire regarding the cursor control and robot control were used. In Figure 10, the results of the NASA-TLX are presented. The able-bodied subjects rated their subjective perceived workload during the procedure of the use-case as low on all six sub-scales (all rating scores < 50; see Figure 10a). The able-bodied subjects gave the highest score for mental demand (rating score: 39.00 ± 21.71 SD) and the lowest score for temporal demand (15.00 ± 8.17 SD).

The subject with tetraplegia showed a similar subjectively perceived workload as the able-bodied subjects (see Figure 10b). The rating scores for the mental, physical, and temporal demand, as well as effort and frustration, were less than 50. Only the own performance was rated poorer by the subject with tetraplegia (rating score: 80.00).

**Figure 11:**
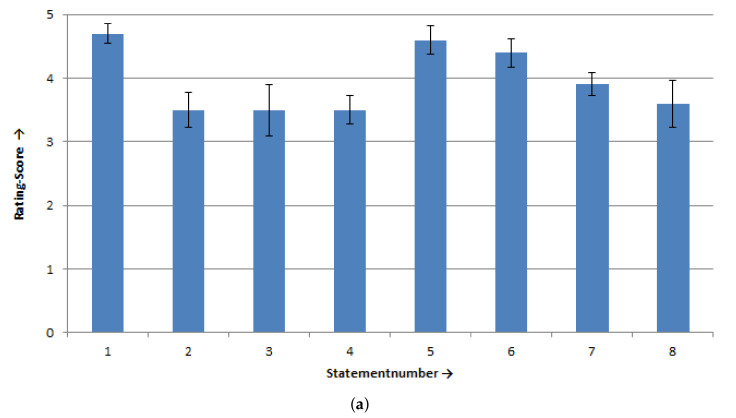

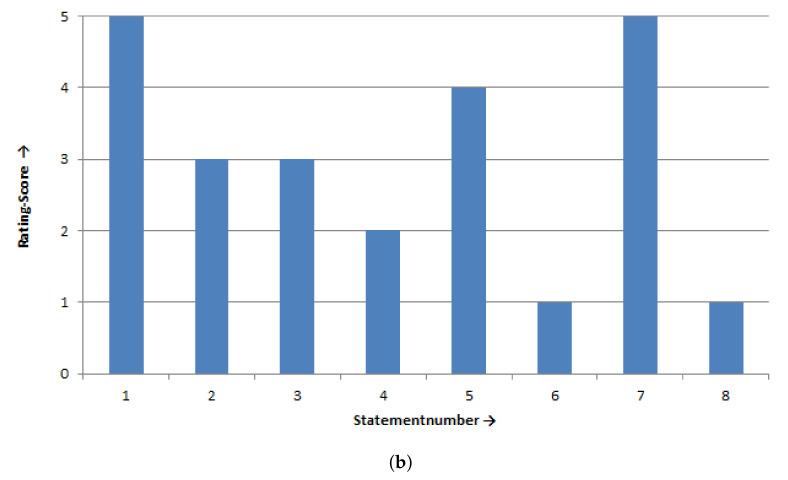
Results of the subjective questionnaire for the use-case, scale from 1 “I do not agree at all” to 5 “I totally agree”: (**a**) able-bodied subjects, mean values ± SE of the rating scores of the eight statements; and (**b**) subject with tetraplegia, values of the rating scores of the eight statements.

Figure 11 depicts the results of the questionnaire in which the control unit was evaluated. The statements of this questionnaire are presented in Table 2. In general, the able-bodied subjects were satisfied with the multimodal unit control (see Figure 11a; all rating scores ≥ 3.5) and they performed the use-case task successfully. The able-bodied subjects considered the cursor GUI and the robot GUI to be clearly arranged and visually appealing (rating scores for Statement 1: 4.70 ± 0.48 SD; and Statement 5: 4.60 ± 0.70 SD). The feedback on the current head position was useful (rating score for Statement 6: 4.40 ± 0.70 SD) and the able-bodied subjects could determine the position of the gripper (rating score for Statement 7: 3.90 ± 0.0.57 SD). The second lowest value of agreement was given by the able-bodied subjects for the evaluation of the easiness of switching between head control and eye control (rating score for Statement 8: 3.60 ± 1.18 SD). The section of the button activation (cursor control) was defined by the Statements 2–4. The able-bodied subjects gave, for these statements, the lowest agreement compared to the other statements (rating scores: 3.50 ± 0.86 SD, 3.50 ± 0.85 SD, and 3.50 ± 0.71 SD, respectively). The button activation was not so easy for the able-bodied subjects.

The subject with tetraplegia gave similar agreements as the able-bodied subjects (see Figure 11b). The cursor GUI and robot GUI were considered visually appealing (rating scores for Statement 1: 5.00 and Statement 5: 4.00) and the subject with tetraplegia could easily determine the gripper position and movement direction (rating score for Statement 1: 5.00). The button activation was also not so easy for the subject with tetraplegia (rating scores: 3.00, 3.00, and 2.00, respectively). The lowest agreement was given for Statements 6 and 8 (both ratings scores: 1.00=). For the subject with tetraplegia, the feedback of the head position was not useful and switching between head control and eye control was very difficult.

## 4. Discussion

In this work, the developed multimodal control unit was evaluated while using it for discrete control (activating of buttons) and for continuous cursor control, in order to discern which modality for which control mode was the best. The hypotheses that the eye tracker would achieve better results for discrete control as well as for continuous cursor control were not confirmed, considering the results of the able-bodied subjects. With both modalities, the subjects showed similar performance; however, the frustration, effort, and error rate were higher while using the eye tracker.

The following points may be the reasons the activation of the buttons was more frustrating and strenuous while using the eye tracker. During fixation, the eyes continuously perform small movements, although the person has the feeling that his gaze is fixed upon something completely calmly. These micro-movements of the eyes are summarized as *microsaccadic jitter* and can be divided into three groups [26,27]. Slow micromovements, also called drift, occur during inter-saccadic intervals. Microsaccades are small and fast movements of eye position (distinguishable from drift movements due to their high speed). Microtremors are irregular and wavelike movements (high frequency and low amplitude). Micromovements may have caused the cursor to move out of the button area during fixation on the button when using the eye tracker. Due to this, the counter in the Dwell Time mechanism was restarted and the activation of a button took longer. Improving fixation detection is a possibility to reduce the influence of microsaccadic jitter. For this purpose, the online visual fixation detection algorithm of Salvucci and Goldberg [28] could be used. The algorithm uses spatial motion and duration thresholds to define a set of allowed pupil position differences between two sequential eye camera images. The spatial motion is the sum of the differences between successive pupil positions, using the eye tracker pixel positions of the eye camera. The spatial motion is compared with the threshold value of the maximum spatial motion. A fixation is detected if the calculated spatial motion stays below the threshold value. The online visual fixation detection algorithm was successfully tested with our kind of eye tracker by Wöhle and Gebhard [21].

The subject’s gaze point was displayed on a monitor located at a distance of 90 cm from the subject. An inaccuracy in pupil detection could have led to greater inaccuracy in the displayed gaze point on the screen, due to this distance. Fixing the eye tracker with an eyewear strap should prevent the eye tracker from moving and, thus, also prevent inaccurate pupil detection; however, it does not completely prevent this effect.

The subject with tetraplegia, due to the disease pattern, was more limited in head movements than the able-bodied subjects. This might be a reason for the longer activation times for the subject with tetraplegia when using head motions. To verify this hypothesis, repeating the *Activation of Button Test* with more subjects with tetraplegia will be performed in a further study. One reason for the higher temporal demand of the subject with tetraplegia while using the eye tracker for activating the buttons might be the “Midas Touch” problem. To reduce the “Midas Touch” problem, the Dwell Time solution was used in this work; however, the chosen Dwell Time was not suitable for the subject with tetraplegia and should be longer or shorter. Repeating the activation test with different Dwell Times will be carried out, in order to examine this aspect. Another possibility to reduce the “Midas touch” problem is to use a further modality to trigger an action (button activation). In a further study, an EMG signal of the lateral eye muscles could be recorded. With a specific eye movement, e.g., blinking, an action could be triggered.

For the Fitts’s Law test, the option *Randomize Target Conditions* was chosen. This option was to ensure that the order of the six conditions per subject was randomized. Thus, the six conditions were not performed in the same order for each subject and a possible learning effect should have been prevented. An analysis of the frequencies of the six conditions showed, however, that, when performing with the eye tracker, Condition *1* was performed by eight subjects at the beginning (i.e., at the first, second, or third position). In contrast, when performed with the MARG-sensor, only four subjects performed Condition *1* at the beginning. This might provide a possible explanation for the fact that the subjects achieved a significantly lower throughput with the eye tracker only for this condition than with the MARG-sensor. The subjects would have a lower throughput due to a learning effect, and not due to the modality. The randomization process for the Fitts’s Law test only partially worked.

In the *Use-Case Test*, our multimodal control system, consisting of the MARG-sensor and eye tracker, was tested for its usability. Both the robot control with the MARG-sensor and cursor control with the eye tracker, as well as the resulting fixed programmed robot movements (skills), were successfully performed by all subjects. The task of the use-case scenario was solved and the new control unit enabled an aid to drinking.

The low subjective perceived workload indicates that the combination of the modalities (i.e., the MARG-sensor and eye tracker) can be used to control a robot to assist in a drinking task without leading to increased effort and strain in subjects. In the evaluation of the control unit, the approval rate of the subjects indicated that there is potential for improvement in the cursor control, especially regarding the specific button activation. In the use-case scenario, the eye tracker was used for button activation. Due to micro-movements of the eyes which occur during fixation [26,27], the control of the cursor by the eye tracker led to small cursor movements, which were unwanted by the subjects. This made it difficult for the subjects to move the cursor in a controlled manner. The high number of glasses wearers among the able-bodied subjects may be another reason for the lower agreement of the subjects regarding statements about cursor control, compared to statements about robot control. Wearing glasses has an influence on the accuracy of pupil detection, affecting the accuracy of the cursor movement and, thus, the activation of buttons. To improve the continuous cursor control while using the eye tracker, increasing the effective target size once the target is acquired, the so-called spatial hysteresis, is a common principle. This will reduce the influence of micro eye movements when activating buttons with the eye tracker.

## 5. Conclusions and Future Work

For all subjects, head motions were found to be a robust modality to control the robot in a three-dimensional space. For the ten able-bodied subjects, no significant difference between the modalities was found for the activation time and error rate in the button activation test and for throughput (Fitts’s Law task). However, the button activation with the eye tracker was mentally more demanding, strenuous, and frustrating. In addition, the error rate of the Fitts’s Law task was significantly higher with the eye tracker. The subject with tetraplegia activated the buttons more quickly through eye motions, compared to head motions. For the subject with tetraplegia, both modalities showed similar results for throughput; however, the error rate was significantly higher when using the eye tracker. Regarding the overall effectiveness, especially regarding the robustness, represented by the error rate, the MARG-sensor provided better results than the eye tracker. Nine of the ten able-bodied subjects and the subject with tetraplegia performed the task in the drinking use-case scenario without errors. These subjects were able to control the robot arm with a gripper successfully and intuitively with the combination of an eye tracker and a MARG-sensor. When performing the task, all subjects showed a low subjective perceived workload. The developed multimodal control unit, thus, can provide support while drinking. However, the switching between the head control and eye control was not easy for the subject with tetraplegia.

The observed results of the subject with tetraplegia will be verified in a further study. For our future work, an evaluation of the proposed system with a larger number of subjects with tetraplegia is planned. In addition, it is planned to compare the proposed control system with conventional methods. The button activation with the eye tracker has potential for improvement in terms of increasing the effective target size once the target is acquired. In future work, the so-called spatial hysteresis will be implemented. The improvement of the switching between head and eye control, as well as the improvement of the button activation, is an important aspect for future research. Especially the user feedback information, whether the head control or the eye control is active, should be improved. Extension by a further modality is planned, in order to reduce the Midas Touch problem occurring while using the eye tracker. A specific EMG signal of the lateral eye muscles will also be used to trigger a button click.

## Figures and Tables

**Table 1:** The six conditions for the Fitts’s Law test.

Condition No.	Distance (Amplitude A) [Pixel]	Diameter (Width W) [Pixel]
1	600	60
2	600	80
3	600	100
4	800	60
5	800	80
6	800	100

**Table 2:** Statements of the subjective questionnaire about the control unit.

Topic	Number	Statement
*Cursor control*
	1	The graphical user interface is visually appealing and clearly arranged.
	2	It is easy to move the cursor in a controlled way.
	3	It is easy to activate the Dwell buttons.
	4	It is easy for me to activate a specific button.
*Robot control*
	5	The graphical user interface is visually appealing and clearly arranged.
	6	Feedback on the current head position is easy to understand and useful.
	7	It is easy for me to put myself in the gripper and determine the position and movement direction of the gripper.
*General control*
	8	It is easy for me to switch between head control and eye control.

## Abbreviations

The following abbreviations are used in this manuscript:

SCI	Spinal Cord Injury
ADL	Activity of Daily Living
HMI	Human-Machine Interface
SMI	SensoMotoric Instruments
ETG	Eye Tracking Glasses
EEG	Electroencephalography
EMG	Electromyography
AMiCUS	Adaptive Head Motion Control for User-friendly Support
MEMS	Micro-Electro-Mechanical System
MARG	Magnetic AngularRate Gravity
SOC	System on Chip
NASA-TLX	NASA Task Load Index
DOF	Degree of Freedom
GUI	Graphical User Interface
SD	Standard Deviation
ET	Eye Tracker
RTLX	Raw Task Load Index
SE	Standard Error
